# Biomedical Insights of Human Genetic Diversity in Complex Diseases

**DOI:** 10.1155/2015/974809

**Published:** 2015-03-09

**Authors:** M. Esther Esteban, Analabha Basu, Carla M. Calò, Pedro Moral

**Affiliations:** ^1^Department of Animal Biology, Anthropology Section, Faculty of Biology, University of Barcelona, Avinguda Diagonal 643, 08028 Barcelona, Spain; ^2^Biodiversity Research Institute, University of Barcelona, Avinguda Diagonal 643, 08028 Barcelona, Spain; ^3^National Institute of Biomedical Genomics, Netaji Subhas Sanatorium (NSS) (T.B. Hospital), 2nd Floor, Kalyani, West Bengal 741 251, India; ^4^Department of Life and Environmental Sciences, Section of Anthropological Sciences, University of Cagliari, Cittadella Universitaria, SS 554 (km 4,5), Monserrato, 09042 Cagliari, Italy

Once some of the major goals in the understanding of human gene diversity are attained, we have now the opportunity to apply them to the field of biomedicine. The knowledge of complete genomes allowed us to go more deeply into the genetic factors of many diseases. Much effort has been devoted to unravel the genetic and environmental contribution to complex diseases, not without difficulty because they represent the extreme manifestation of a continuum of genetic, physiological, and environmental features. With the added pressure that some of these disorders have reached epidemic proportions because of the lifestyle changes of worldwide populations.

Within this scenario, this special issue contains five works about complex diseases, including heart pathologies, rheumatoid arthritis, colorectal cancer, and childhood asthma. All are multistep diseases with unknown interactions among environmental agents and genetic susceptibility. In addition, their incidences do not stop increasing in both western and emerging countries. The methodological approximations of the five articles respond to different designs including miRNAs binding sites, mitochondrial heteroplasmy, SNP genotyping, and a very particular sample. In the paper entitled “Mosaicism of Mitochondrial Genetic Variation in Atherosclerotic Lesions of the Human Aorta,” the samples are segments of morphologically mapped aortic walls instead of human patients as in the remaining papers. In this work, M. A. Sazonova et al. examine the heteroplasmy levels of eleven mitochondrial mutations in segments of morphologically mapped aortic walls, normal and affected by atherosclerosis segments of morphologically mapped aortic walls. Five mutations have been found significantly associated with atherosclerotic lesions of intimal segments.

The next paper concerning heart diseases is entitled “*Novel Mutations* in the Transcriptional Activator Domain of the Human TBX20 in Patients with Atrial Septal Defect.” The work conducted by I. E. Monroy-Muñoz et al. analyzes patients affected by congenital heart defects for TBX20 mutations. Three missense mutations located in exons encoding the transcriptional activator domain have been detected, together with other ten nonsense mutations and one nonreported SNP.

The work entitled “*TRAF1/C5* but Not* PTPRC* Variants Are Potential Predictors of Rheumatoid Arthritis Response to Antitumor Necrosis Factor Therapy” examines the association between risk variants of rheumatoid arthritis and response to treatment with antitumor necrosis factor. In their paper, H. Canhão et al. examine more than six hundred Portuguese and Spanish patients and fail to replicate the previously reported association of* PTPRC* locus and response to treatment.

The relationship among risk of asthma, environmental exposures, and genetic background has been deeply examined in the paper entitled “Gender-Dependent Effect of GSTM1 Genotype on Childhood Asthma Associated with Prenatal Tobacco Smoke Exposure.” C.-C. Wu et al. conducted a longitudinal birth cohort study of six years of follow-up recruiting more than five hundred children to explore the interactive influences of gender, GSTM1 genotypes, and prenatal tobacco smoke exposure in asthma development.

Finally, the paper entitled “A Functional Variant at miR-520a Binding Site in PIK3CA Alters Susceptibility to Colorectal Cancer in a Chinese Han Population” investigates the association between miR-520a binding site polymorphism in the PIK3CA gene and risk of colorectal cancer. L. Ding et al. conclude that polymorphisms in untranslated regions of PIK3CA may play a role in colorectal carcinogenesis.

